# Awake Insights for Obstructive Sleep Apnea: Severity Detection Using Tracheal Breathing Sounds and Meta-Model Analysis

**DOI:** 10.3390/diagnostics16030448

**Published:** 2026-02-01

**Authors:** Ali Mohammad Alqudah, Zahra Moussavi

**Affiliations:** 1 Biomedical Engineering Program, University of Manitoba, Winnipeg, MB R3T 5V6, Canada; zahra.moussavi@umanitoba.ca; 2 Department of Electrical and Computer Engineering, University of Manitoba, Winnipeg, MB R3T 5V6, Canada

**Keywords:** obstructive sleep apnea, OSA severity prediction, tracheal breathing sounds, wakefulness screening, meta-models

## Abstract

**Background/Objectives:** Obstructive sleep apnea (OSA) is a prevalent, yet underdiagnosed, disorder associated with cardiovascular and cognitive risks. While overnight polysomnography (PSG) remains the diagnostic gold standard, it is resource-intensive and impractical for large-scale rapid screening. **Methods:** This study extends prior work on feature extraction and binary classification using tracheal breathing sounds (TBS) and anthropometric data by introducing a meta-modeling framework that utilizes machine learning (ML) and aggregates six one-vs.-one classifiers for multi-class OSA severity prediction. We employed out-of-bag (OOB) estimation and three-fold cross-validation to assess model generalization performance. To enhance reliability, the framework incorporates conformal prediction to provide calibrated confidence sets. **Results:** In the three-class setting (non, mild, moderate/severe), the model achieved 76.7% test accuracy, 77.7% sensitivity, and 87.1% specificity, with strong OOB performance of 91.1% accuracy, 91.6% sensitivity, and 95.3% specificity. Three-fold confirmed stable performance across folds (mean accuracy: 77.8%; mean sensitivity: 78.6%; mean specificity: 76.4%) and conformal prediction achieved full coverage with an average set size of 2. In the four-class setting (non, mild, moderate, severe), the model achieved 76.7% test accuracy, 75% sensitivity, and 92% specificity, with OOB performance of 88.2% accuracy, 91.6% sensitivity, and 88.2% specificity. **Conclusions:** These findings support the potential of this non-invasive system as an efficient and rapid OSA severity assessment whilst awake, offering a scalable alternative to PSG for large-scale screening and clinical triaging.

## 1. Introduction and Literature Review

Obstructive sleep apnea (OSA) is highly prevalent yet substantially underrecognized; in Canada, nearly one quarter of adults are estimated to be at high risk, with most cases undiagnosed [1,2]. The economic burden in the United States alone exceeds $150 billion annually [3], underscoring the need for scalable and rapid assessment strategies.

Overnight polysomnography (PSG) remains the diagnostic gold standard but is resource-intensive and often inaccessible due to long wait times [4]. Thus, simpler overnight tools, in practice, simpler overnight tools such as fingertip oximetry are widely used for home triage. The oxygen desaturation index (ODI) derived from nocturnal SaO_2_ [5] correlates strongly with the apnea–hypopnea index (AHI) and can classify moderate-to-severe OSA with good accuracy [6]. However, these methods depend on capturing sleep-related desaturation events, making them unsuitable for time-sensitive situations, i.e., surgeries requiring general anesthesia that need a quick and accurate assessment [7].

Wakefulness-based detection offers a complementary approach by identifying anatomical and physiological traits of airway collapsibility without requiring sleep monitoring [8,9]. This is particularly valuable in perioperative care, where a quick risk stratification is critical for preventing complications [10], and in outpatient or emergency settings, where PSG or overnight testing is not feasible. Questionnaires such as STOP-Bang provide high sensitivity but limited specificity, especially in obese patients [11,12,13,14]; thus, the need for accurate, non-invasive wakefulness-based methods is greatly of interest.

Recent advances in biological signal processing have shown that tracheal breathing sounds (TBS) recorded during wakefulness can provide a rapid, low-resource screening and risk stratification approach for OSA severity, particularly when overnight sleep monitoring is impractical [9]. When combined with anthropometric information, TBS-based models have demonstrated clinically relevant accuracies at treatment thresholds [8]. These findings suggest that wakefulness detection may bridge the gap between resource-intensive PSG and simple but nonspecific tools like STOP-Bang or overnight oximetry, enabling rapid, point-of-care identification of at-risk patients with OSA.

Several studies have shown the promise of TBS recorded during wakefulness as a low-cost, non-invasive alternative to overnight testing [8,9,15,16]. An initial work in small samples showed an accuracy over 90% accuracy in distinguishing severe OSA from non-OSA using spectral and nonlinear features of TBS [9]. Later, by combining TBS with anthropometric measures, it was shown that OSA could be detected robustly by clinically relevant accuracy in a much larger samples [8]. More recent work has confirmed the feasibility of advanced acoustic features and machine learning in improving robustness of the technique [17]. Collectively, these findings establish TBS as one of the most promising wakefulness-based tools for rapid OSA assessment, complementing, but fundamentally distinct from, overnight SaO_2_ monitoring [5,6]. Nevertheless, all the existing techniques have been limited to binary detection of OSA versus non-OSA.

Unlike prior wakefulness-based TBS studies [8,9,15,16], which primarily focused on binary OSA detection or single clinical thresholds, the present work addresses the more challenging task of multi-class severity stratification. Moreover, previous studies reported point estimates without uncertainty quantification, limiting clinical interpretability. By integrating pairwise acoustic specialization with conformal prediction, the proposed framework provides both severity stratification and calibrated confidence sets, addressing a key gap in prior TBS-based OSA research.

In this study, we present a framework for multi-class OSA severity screening and risk stratification during wakefulness, intended to complement rather than replace overnight diagnostic testing. Building on previously developed wakefulness-based TBS classifiers, the proposed framework integrates pairwise acoustic specialization within a stacked meta-model architecture. By incorporating conformal prediction, the system enables multi-level severity stratification with calibrated uncertainty estimates, supporting rapid and uncertainty-aware point-of-care triage and addressing a key gap in existing wakefulness-based OSA screening approaches.

## 2. Materials and Methods

This study presents a meta-model framework designed to classify OSA severity into four clinically relevant categories: Non-OSA, Mild, Moderate, and Severe. Recognizing the acoustic and physiological overlap between adjacent severity levels, the framework adopts a modular structure composed of six binary base classifiers trained in our earlier work [18], each targeting one-vs.-one class distinctions (e.g., Non-OSA vs. Mild, Mild vs. Moderate).

**Figure 1:**
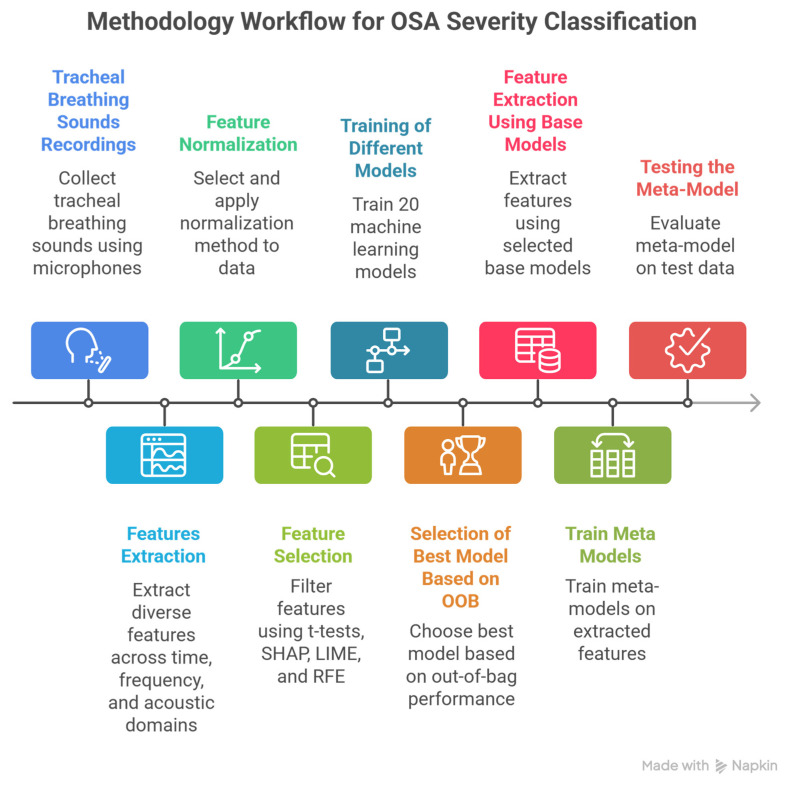
General steps of the proposed model.

This work extends and completes the methodology introduced in [18] by unifying these previously developed binary classifiers into a single stacked meta-model that enables end-to-end multi-class OSA severity prediction. In this work, we reuse these classifiers and their associated feature sets to train a stacked ensemble meta-model capable of multi-class prediction. To enhance clinical applicability, the framework integrates conformal prediction, which augments predictions with calibrated uncertainty estimates. The complete system architecture and data flow are illustrated in Figure 1.

### 2.1. Dataset

The dataset used in this study was adopted from our previous work [18] which includes TBS recordings and anthropometric data from 199 individuals (74 non-OSA, 35 Mild-OSA, 50 Moderate-OSA, and 40 Severe-OSA). OSA severity groups were defined based on apnea–hypopnea index (AHI) values as follows: Non-OSA: AHI < 5 events/h, Mild OSA: 5 ≤ AHI < 15 events/h, Moderate OSA: 15 ≤ AHI < 30 events/h, and Severe OSA: AHI ≥ 30 events/h. Breathing sounds were recorded while participants were awake (daytime) in supine position using a Sony ECM77B microphone, Tokyo, Japan omnidirectional condenser microphone (sensitivity: −52 dB ± 3.5 dB, frequency response: 40 Hz–20 kHz). placed over the suprasternal notch. Table 1 presents the total number of subjects in each severity group and their corresponding anthropometric data for the dataset used in this study. Study participants were instructed to breathe deeply for five full breath cycles through their nose, followed by another 5 deep breath cycles through their mouth with a nose clip in place. The study was approved by the Biomedical Research Ethics Board of the University of Manitoba, and all study subjects provided informed consent prior to participation.

### 2.2. Dataset Split and Cohort Stratification for Training and Testing

To evaluate the model’s generalization capability, the dataset comprising 199 subjects was split into 85% for training (*n* = 169) and 15% for testing (*n* = 30). To minimize training bias, the model was trained 10 times, each with a shuffled version of the dataset. Stratified sampling was used to ensure consistent distribution of clinical and demographic variables across both sets. Specifically, stratification preserved the distributions of OSA severity classes and key anthropometric variables, including age, body mass index (BMI), neck circumference (NC), and Mallampati score (MPS). The training subset was used for feature selection, model training, and internal validation via out-of-bag (OOB) estimates. The held-out test set remained untouched until final evaluation to provide an unbiased measure of real-world performance. Table 2 and Table 3 summarize the anthropometric profiles of subjects across severity groups in both subsets.

This stratified dataset split preserves clinical relevance and demographic diversity, enabling robust training and unbiased final performance reporting. Such careful design is crucial in sleep apnea research, where severity overlaps and physiological variance can challenge model generalization.

### 2.3. Dataset Split and Cohort Stratification for K-Fold Cross Validation

Standard stratified k-fold cross-validation was unsuitable for this study because it could not maintain consistent stratification across both OSA severity and key anthropometric factors, particularly within smaller severity classes. To address this, a custom stratified k-fold method was developed to balance folds across severity groups and major anthropometric thresholds (age (<50 vs. ≥50), BMI (<35 vs. ≥35), neck circumference (≤40 vs. >40), sex (male vs. female), and Mallampati scores). This approach preserved the joint distribution of clinically relevant subgroups, reduced bias, and produced more representative and reliable training and testing sets, especially for underrepresented samples. Table 4 summarizes the anthropometric distribution across the folds.

### 2.4. Preprocessing and Feature Engineering

All audio recordings were preprocessed using a pipeline that included signal denoising, phase segmentation using LogVar, and fourth-order Butterworth bandpass filtering (75–3000 Hz), as detailed in our previous work [18]. The resulting inspiratory and expiratory segments were then processed using a comprehensive feature extraction and selection framework, also introduced in [18], designed to capture both linear and nonlinear signal characteristics. Extracted features included spectral descriptors (e.g., power spectral density, spectral entropy), higher-order spectral features (e.g., bispectrum, bicoherence), time-domain measures (e.g., fractal dimension, peak statistics), and time–frequency representations using 4-level Symlet4 wavelet and constant-Q transforms [18]. Additional cross-domain features such as recurrence quantification, Lyapunov exponents, and perturbation metrics (jitter, shimmer) were included to provide a multidimensional view of breathing dynamics [18]. In parallel, Anthropometric data, including age, BMI, NC, and MPS, were processed using the imputation strategy proposed in [18]. Missing values were imputed using a stratified k-nearest neighbors (k-NN) approach with k = 3, with subjects grouped by OSA severity (non-OSA, mild, moderate, severe) to preserve group-specific distributions and reduce bias [18].

Following feature extraction and imputation, adaptive normalization was performed as described in [18]. Four normalization techniques, z-score, min-max, mean-range, and robust scaling, were evaluated for each feature, and the method that maximizes mutual information with class labels was selected. This ensured consistent feature scaling while preserving discriminative patterns and enhancing robustness to outliers.

To further refine the dataset, a three-stage feature selection pipeline was applied, as proposed in [18]. Initially, univariate *t*-tests were used to remove non-significant features. Remaining features were ranked using SHAP (Shapley Additive exPlanations) values from an ensemble classifier. Finally, recursive feature elimination (RFE) with a RUSBoost classifier was employed, guided by cross-validation, to select the most impactful features [19]. This pipeline ensured optimal model performance by retaining only stable and informative features. The total number of selected features for each model is 35:30 selected using the feature selection pipeline and 5 from the anthropometric data.

### 2.5. Base Models

Six one-vs.-one binary base models were developed for pairwise OSA severity classification, as proposed in our previous framework [18] targeting the following comparisons:

- Non-OSA vs. Mild-OSA;
- Non-OSA vs. Moderate-OSA;
- Non-OSA vs. Severe-OSA;
- Mild-OSA vs. Moderate-OSA;
- Mild-OSA vs. Severe-OSA;
- Moderate-OSA vs. Severe-OSA.

Each model was optimized using Bayesian hyperparameter tuning across 20 candidate classifiers, including Support Vector Machine (SVM), logistic regression, random forests, neural networks, and RUSBoost. Bootstrap aggregation with out-of-bag (OOB) validation was employed to enhance model stability and generalization. Each base model outputs a posterior probability (or score) for its respective binary classification, as outlined in [18].

### 2.6. Meta-Model Construction

**Figure 2:**
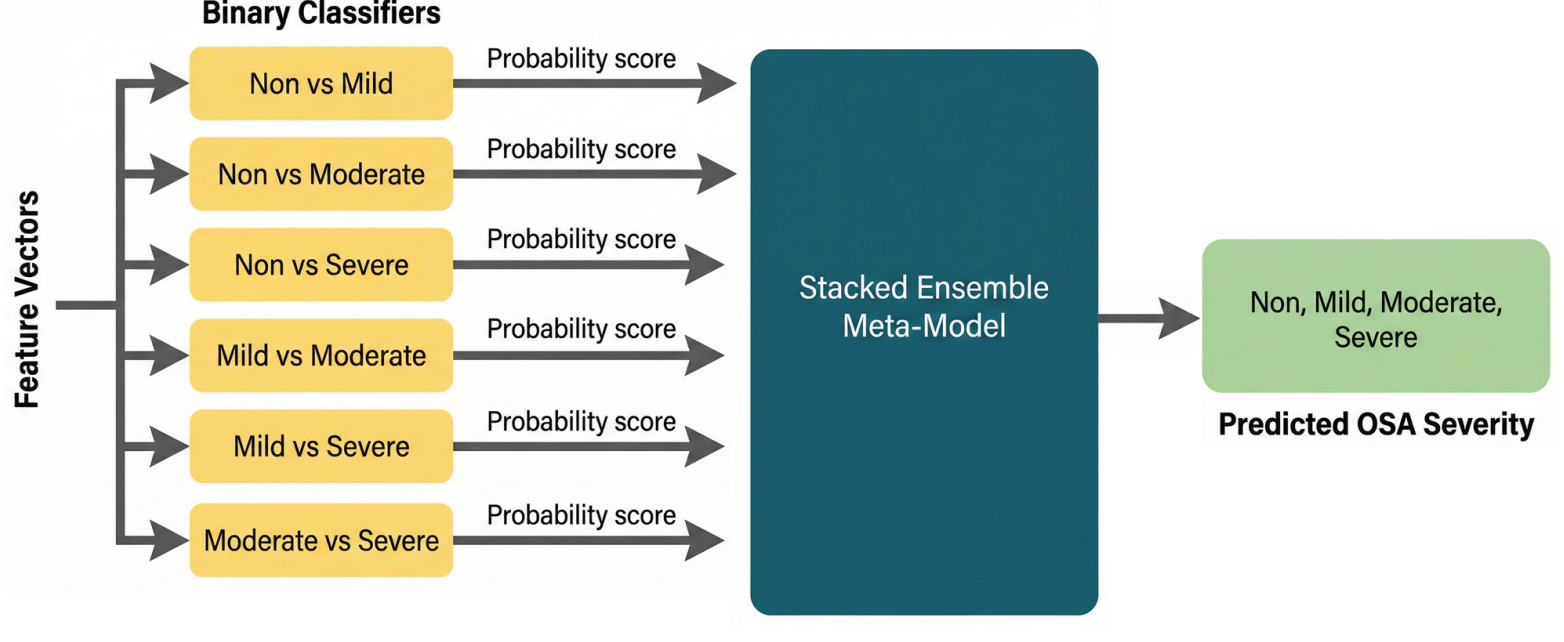
Meta-model for multi-class OSA severity classification.

The 12 prediction scores (i.e., probability outputs) from the base models were used as input features for the meta-model. This stacked approach allows the meta-model to learn higher-level patterns from the decision landscape of individual base models, capturing interactions across all severity levels [20]. Multiple meta-classifiers were evaluated, including: SVM with radial and polynomial kernels, Random Forests, Logistic Regression, and Multi-layer Perceptron (MLP) [21]. The final meta-model was selected based on its performance on a blind test set (15% of the dataset) or the testing fold in the k-fold cross- validation, which was stratified to ensure class balance. The meta-models are trained for the three classes (Non-OSA, Mild-OSA, and Moderate/Severe-OSA) and four classes (Non-OSA, Mild-OSA, Moderate, and Severe-OSA). Each classification problem was treated independently, with the complete training methodology applied to every pairwise comparison. This ensures that base classifiers are explicitly optimized for the discriminative characteristics relevant to each subset of the data. Figure 2 shows the block diagram of the meta-model used.

### 2.7. Conformal Prediction

To enhance the reliability of OSA severity classification, we incorporate a conformal prediction (CP) framework that augments standard probabilistic classifiers with principled uncertainty quantification. CP constructs prediction sets that, under mild assumptions (notably, the exchangeability of calibration and test data), offer guaranteed coverage as a critical feature in risk-sensitive domains like clinical diagnostics [22]. Also, this framework provides a transparent mechanism to communicate predictive uncertainty, which is crucial in clinical applications where misclassification can affect patient management.

**Figure 3:**
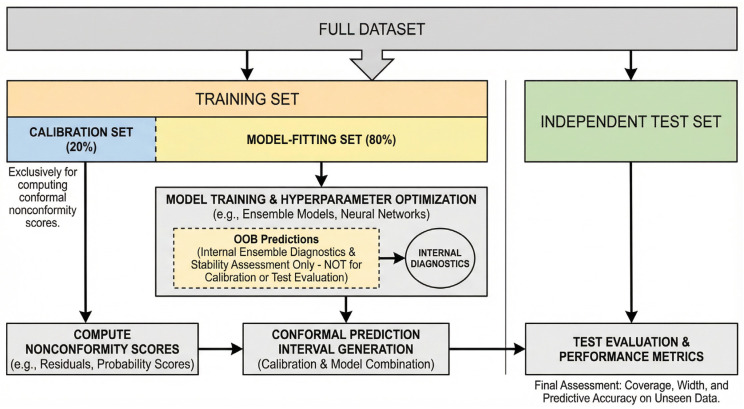
Conformal prediction workflow for OSA severity classification. The training set is split into a calibration set (20%) for computing nonconformity scores and a model-fitting set (80%) for ensemble training. OOB predictions are used only for internal diagnostics and are not involved in calibration or testing, ensuring proper separation and reliable uncertainty estimation.

The training set is partitioned into two subsets: an 80% model-fitting set, used for training and hyperparameter optimization, and a 20% calibration set, used exclusively to compute nonconformity scores. Out-of-bag (OOB) predictions from ensemble base classifiers (e.g., random forests or gradient boosting) are computed solely for internal diagnostics and stability assessment; they are not used for conformal calibration, model selection, or final evaluation. Figure 3 illustrates the conformal prediction workflow for OSA severity classification, including the training–calibration–test split and the role of OOB predictions.

For a given significance level α∈(0,1), the conformal predictor generates a prediction $C(x_{\mathrm{test}})\subseteq Y$ for each test instance $x_{\mathrm{test}}$ [23], such that

(1) $$P\left(Y_{\mathrm{test}}\in C\left(x_{\mathrm{test}}\right)\right)\geq 1-\alpha$$

This provides a high-confidence region where the true class label is expected to reside, without making strong distributional assumptions [22,23]. Then, to quantify the “strangeness” of potential label assignments, we define a nonconformity score as follows:

(2) $$s(x,y)=1-\hat{p}(y|x)$$

where $\hat{p}(y|x)$ denotes the estimated class probability. In our implementation, this probability is derived through ensemble averaging using out-of-bag (OOB) predictions from base classifiers (e.g., random forests or gradient boosting models), ensuring reliable and unbiased estimates [22,24]. After the initial train–test split or k-fold separation, the training data are further partitioned using a hold-out strategy to create an independent calibration set for conformal prediction. Specifically, 20% of the training samples are randomly held out using MATLAB (R2024b)’s cvpartition function (HoldOut = 0.2), denoted $D_{\mathrm{calib}}=\{(x_{i},y_{i}){\}}_{i=1}^{n}$. For each calibration sample, the nonconformity score $\alpha_{i}=s(x_{i},y_{i})$ is calculated [22,24]. The inclusion threshold was determined by computing the empirical quantile:

(3) $$\hat{q}={\mathrm{Quantile}}_{1-\alpha }(\{\alpha_{i}{\}}_{i=1}^{n}\cup \{\infty \})$$

Then, for each test instance $x_{\mathrm{test}}$. The prediction set was defined as follows:

(4) $$C\left(x_{\mathrm{test}}\right)=\left\{y\in Y|s\left(x_{\mathrm{test}},y\right)\leq \hat{q}\right\}$$

The conformal framework ensures that the prediction set contains the actual OSA severity class with probability at least 1−α provided that the calibration and test data are exchangeable [22,24]. The prediction set size is adaptively determined: smaller sets indicate higher model confidence, which is particularly important when distinguishing between clinically adjacent severity categories, such as moderate and severe OSA.

The independent test set remains completely untouched until the final performance evaluation, preserving the finite-sample coverage guarantees of CP under the exchangeability assumption. High-confidence predictions typically yield a single class, while ambiguous inputs may include multiple labels (e.g., {moderate, severe}), effectively signaling diagnostic uncertainty [22,24]. The adaptive size of the prediction set reflects model confidence, which is particularly important when distinguishing clinically adjacent severity categories.

**Figure 4:**
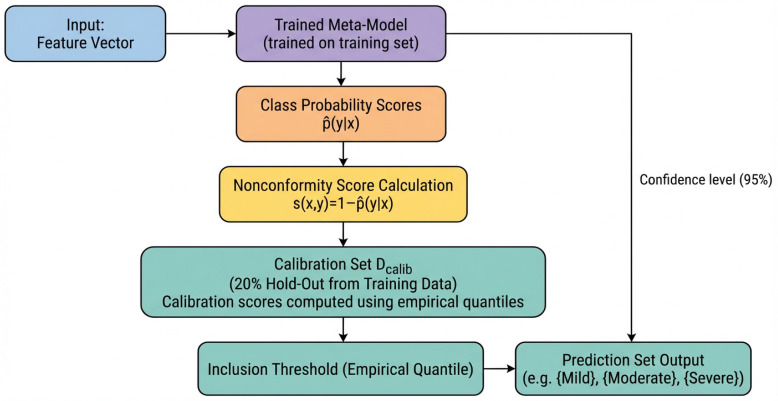
Conformal prediction pipeline for uncertainty-calibrated OSA severity classification.

CP is model-agnostic and can be applied atop any probabilistic classifier, including those already optimized for the task. When combined with ensemble-based models, CP benefits from base-learner diversity and cross-model aggregation, mitigating individual model biases and improving overall reliability. In OSA severity detection, where incorrect classification may lead to inappropriate treatment, CP provides a principled mechanism for communicating uncertainty. For instance, borderline moderate/severe cases may yield a prediction set such as {moderate, severe}, transparently indicating ambiguity and encouraging further clinical evaluation. Figure 4 illustrates how CP is integrated with the proposed meta-model, highlighting its role in generating uncertainty-calibrated predictions that enhance interpretability and clinical safety.

### 2.8. Model Evaluation

The evaluation framework combines traditional classification performance metrics with conformal prediction analysis to comprehensively assess model effectiveness and uncertainty quantification comprehensively. This dual-axis evaluation strategy addresses the reliability of predictive accuracy and confidence estimates.

#### 2.8.1. Evaluation Protocol

- Out-of-Bag (OOB) Validation: During ensemble training, each base learner was evaluated on the subset of training samples excluded from its bootstrap resample. These out-of-bag predictions were used to estimate performance without requiring a separate validation set. The resulting OOB metrics provide an unbiased estimate of generalization error.
- Independent Test Evaluation: After model training, performance was assessed using the same metrics on a held-out test set. To account for randomness in training (e.g., bootstrap sampling or stochastic optimization), the evaluation was repeated over 25 independent trials with different random seeds.

#### 2.8.2. Performance Metrics

Models were evaluated using three core metrics: accuracy, sensitivity, and specificity, measured on both out-of-bag (OOB) samples and independent test sets. These metrics were selected to ensure a balanced assessment of model performance, particularly in the presence of class imbalance [25].

Accuracy reflects the overall proportion of correctly classified instances out of the total number of samples. It provides a general measure of how well the model performs across all predictions, regardless of class [25].

Macro-averaged sensitivity represents the average actual positive rate across all classes. It is calculated by determining the sensitivity (true positives divided by the sum of true positives and false negatives) for each class individually, then averaging the results. This approach ensures that each class contributes equally to the overall sensitivity score, regardless of its frequency in the dataset [25].

Macro-averaged specificity captures the average actual negative rate across all classes. For each class, specificity is calculated by dividing the number of true negatives by the sum of true negatives and false positives. Like macro sensitivity, macro specificity averages these values across all classes to prevent dominance by majority classes. These macro-level metrics provide a more equitable evaluation of model performance in multi-class classification settings, particularly when the class distribution is unbalanced [25].

#### 2.8.3. Conformal Prediction Metrics

Conformal prediction was integrated into the evaluation framework to evaluate the uncertainty quantification capability of the models. The two main metrics were coverage and average set size [22,24]. Coverage, the proportion of instances where the actual label is included in the prediction set:

(5) $$\mathrm{Coverage}=\frac{1}{N_{\mathrm{test}}}\sum_{i=1}^{N_{\mathrm{test}}}I\left(y_{i}\in C\left(x_{i}\right)\right)$$

Average Set Size, which reflects the efficiency of the prediction set:

(6) $$\mathrm{Avg}\;\mathrm{Set}\;\mathrm{Size}\;=\;\frac{1}{N_{\mathrm{test}}}\sum_{i=1}^{N_{\mathrm{test}}}|C\left(x_{i}\right)|$$

These metrics provide insights into the trade-off between prediction confidence and set efficiency [22,24]. Class labels were numerically encoded to preserve categorical structure [22,24]. All performance metrics were averaged across 15 trials to mitigate the impact of stochastic variability. Stats averaged, including classifier rankings and 95% confidence intervals, were computed using non-parametric bootstrap resampling.

## 3. Results

The proposed meta-model was evaluated on a held-out test set to assess its generalization ability across the four-class OSA severity classification task. This evaluation followed a two-stage process: (1) assessing base model aggregation and meta-model performance during training using out-of-bag (OOB) validation, and (2) blind testing on unseen data. Section 4.1 presents the Training–Testing Meta-Model Performance Summary, while Section 4.2 reports the k-fold results across the three validation folds.

### 3.1. Training-Testing Meta-Model Performance Summary

**Figure 5:**
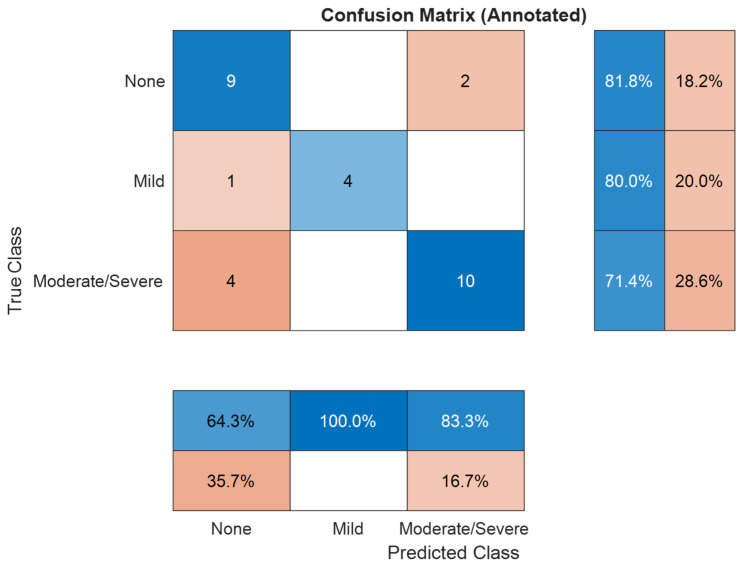
Confusion matrix for three classes of OSA severity classification. Blue indicates correctly classified samples, red indicates misclassifications, with color intensity proportional to the number of samples.

For the three classes of OSA severity problem, as presented in Table 5 and illustrated in Figure 5, the meta-model demonstrated strong performance in the OOB evaluation for three classes using neural network model of two hidden layers with learning rate of 7.06-2, achieving an accuracy of 91.1%, sensitivity of 91.6%, and specificity of 95.3%. When applied to the blind test set, the performance exhibited a moderate decline typical for ensemble frameworks with a final test accuracy of 76.7%, sensitivity of 77.7%, and specificity of 87.1%. This reduction can be attributed primarily to the limited size and non-diversity of the dataset, as well as the constrained feature set used for training. In smaller and less varied datasets, the model’s ability to generalize to unseen data can be restricted, particularly when specific feature patterns are underrepresented. Nevertheless, the meta-model maintained strong generalization capability, especially in preserving high specificity. This robustness is supported by the complementary strengths of the base classifiers and the architecture of the stacking ensemble, which together mitigate the risks associated with dataset limitations.

To further assess prediction reliability, a conformal prediction framework was applied. The conformal model achieved full coverage (1.0), ensuring that each test instance’s prediction set contained the correct class, with an average prediction set size of 2, indicating high confidence in classification outcomes. These results highlight the meta-model’s ability not only to produce accurate predictions but also to quantify the certainty of its decisions, which is particularly important in clinical applications where decision reliability is critical.

The confusion matrix in Figure 5 provides further insight into class-level performance, revealing that the meta-model maintained strong predictive ability across all OSA severity categories, with misclassifications occurring primarily between adjacent severity levels, a common trend in medical classification tasks where class boundaries are gradual rather than discrete. This behavior suggests that the model effectively captures the underlying patterns distinguishing severely from non-severe cases, while borderline cases remain the most challenging to classify.

**Figure 6:**
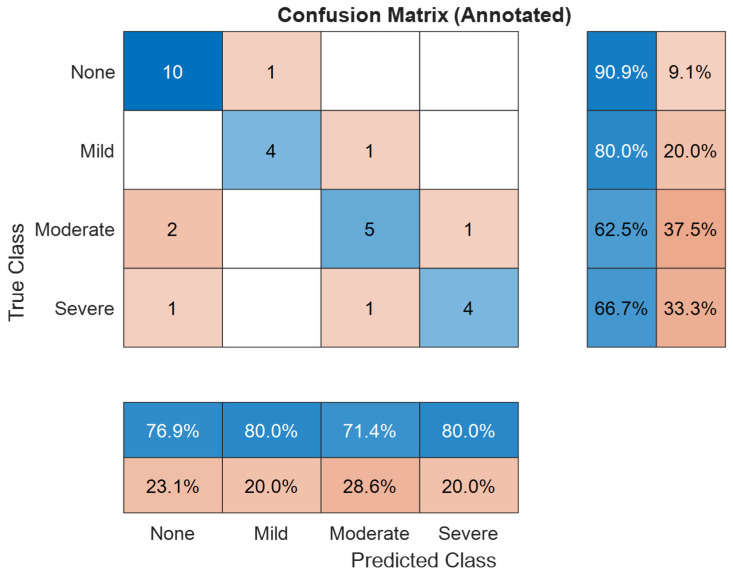
Confusion matrix for four classes of OSA severity classification. Blue indicates correctly classified samples, red indicates misclassifications, with color intensity proportional to the number of samples.

The four classes of OSA severity problem, as shown in Table 6 and illustrated in Figure 6, the meta-models demonstrate a strong performance during the out-of-bag (OOB) evaluation for four classes using neural network model of three hidden layers with learning rate of 5.14-3, with an accuracy of 88.2%, sensitivity of 91.6%, and specificity of 88.2%. On the independent test set, accuracy decreased to 76.7%, while sensitivity and specificity were 75% and 92%, respectively. This decline reflects the limited size and diversity of the dataset, alongside a constrained feature set. Smaller, less varied datasets can restrict the model’s ability to generalize, particularly when specific feature patterns are underrepresented.

Despite these factors, the model preserved robust performance as a proof-of-concept, particularly in terms of specificity, supported by the complementary strengths of the base classifiers and the stacking ensemble framework. Prediction confidence was further assessed using a conformal prediction method, which achieved full coverage ensuring that every test instance’s prediction set contained the true class and an average prediction set size of 2. These results provide preliminary evidence of meaningful certainty in the model’s outputs, an important consideration for future clinical translation.

Figure 6 confusion matrix provides insight into the model’s performance across all four OSA severity levels. Misclassifications mainly occurred between adjacent severity categories, consistent with the gradual nature of medical condition boundaries. This trend indicates that the model effectively distinguishes clear-cut cases, while borderline instances remain more difficult to classify accurately.

**Figure 7:**
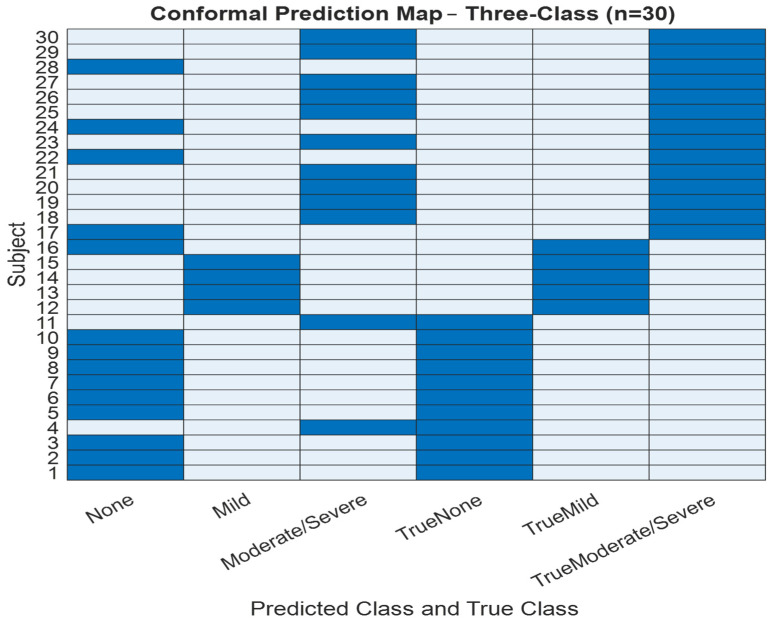
Three-class testing conformal prediction set map.

**Figure 8:**
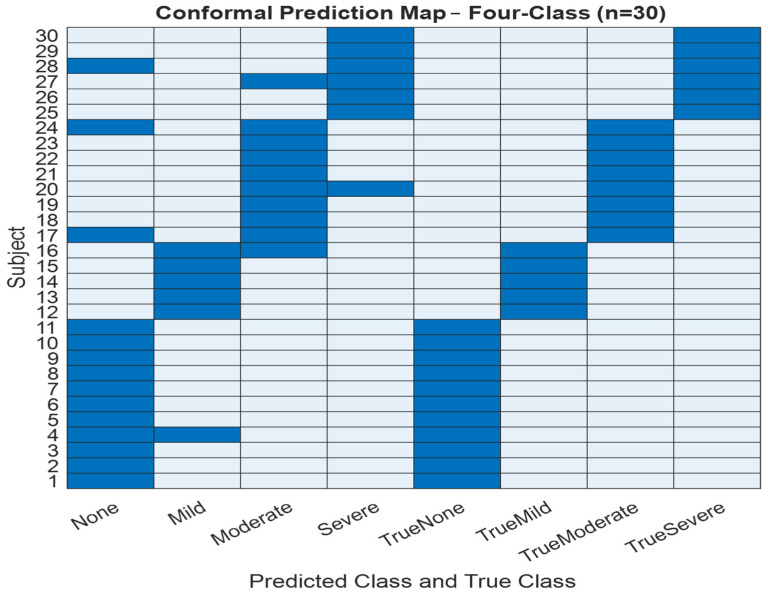
Four-class testing conformal prediction set map.

To improve the interpretability and reliability of classification outputs, we implemented conformal prediction, which generates prediction sets rather than single-label outputs. Figure 7 and Figure 8 present the conformal prediction map for the 30 test subjects for the three and four classes, respectively, illustrating which severity classes were included in each prediction set at a 95% confidence level. Each row corresponds to a subject, and each column corresponds to a possible class label (Non, Mild, Moderate/Severe) for three classes and (Non, Mild, Moderate, Severe) for four classes. A blue cell indicates that the corresponding class was included in the prediction set. For highly confident predictions, the set typically included a single class, whereas uncertain predictions encompassed two or more labels. This visualization confirms that while full coverage (100%) was achieved, the average set size was 2 for three classes and 4 for four classes, indicating increased ambiguity in some cases, particularly between adjacent severity levels. The conformal framework provided interpretable uncertainty quantification, aligning well with the observed performance drop in blind testing and offering a mechanism for flagging diagnostically ambiguous cases for further review.

Table 7 provides a breakdown of the classification performance across the individual OSA severity groups in the test set. The Non-OSA class achieved a balanced profile with 81.8% sensitivity and 73.7% specificity, indicating strong recognition of healthy individuals with minimal false positives.

The Mild-OSA class was classified with 100% specificity and 80.0% sensitivity, suggesting the meta-model was highly conservative when assigning this class, minimizing false positives. However, Mild cases were the most frequently confused group in earlier base models, so this performance reflects a significant improvement in class separation.

The Moderate/Severe OSA group, which had been combined due to overlapping acoustic characteristics, achieved 71.4% sensitivity and 87.5% specificity. This class presented the most significant challenge for precise sensitivity, likely due to intra-group variability and class imbalance.

**Figure 9:**
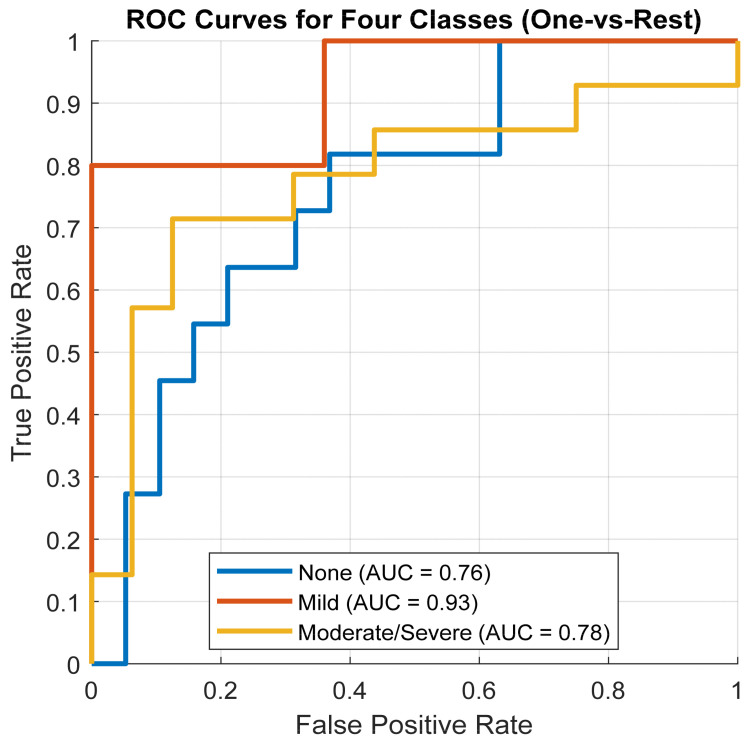
ROC curves and AUC for the three-class testing set.

The ROC curves for the three classes are shown in Figure 9. The Non-OSA group achieved an AUC of 0.76, the Mild-OSA group achieved the highest AUC of 0.93, and the Moderate/Severe-OSA group achieved an AUC of 0.78. These values are consistent with the per-class performance metrics, highlighting the strong separability of the Mild-OSA class and the relatively greater overlap between Moderate/Severe and other classes.

Table 8 summarizes the classification performance across the four individual OSA severity groups in the test set. The Non-OSA class achieved 91% sensitivity and 84% specificity, demonstrating effective identification of healthy individuals, with a moderate false-positive rate due to residual overlap with Mild OSA patterns.

The Mild OSA class was classified with 80.0% sensitivity and 96% specificity, indicating that the meta-model adopted a conservative strategy when assigning this class, resulting in very few false positives. This represents a substantial improvement, as Mild cases were previously the most frequently confused category in the base classifiers.

For the Moderate OSA class, the model reached 63.0% sensitivity and 91% specificity, suggesting a cautious approach with a tendency to avoid false alarms but at the cost of missing some actual cases reflecting a conservative decision boundary that prioritizes specificity over sensitivity. This may be attributed to the class’s acoustic overlap with adjacent severity levels.

The Severe OSA class achieved 68% sensitivity and 96% specificity, reflecting the model’s strong ability to confidently identify high-risk individuals when predicted, while maintaining a low false-positive rate. Although sensitivity remains moderate, the high specificity is clinically valuable for prioritizing patients requiring urgent intervention.

**Figure 10:**
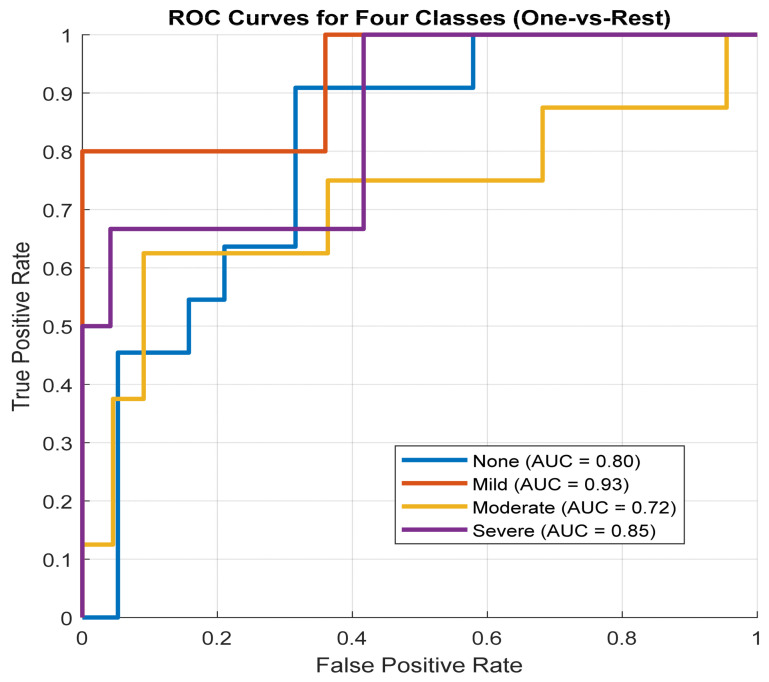
ROC curves and AUC for the four-class testing set.

The ROC curves for the three classes are shown in Figure 10. The Non-OSA group achieved an AUC of 0.80, the Mild-OSA group achieved the highest AUC of 0.93, the Moderate-OSA group achieved an AUC of 0.72, and the Severe-OSA group achieved an AUC of 0.85. These values are consistent with the per-class performance metrics, highlighting the strong separability of the Mild-OSA class and the relatively greater overlap between Moderate/Severe and other classes.

### 3.2. K-Fold Meta-Model Results

The selected base classifiers consistently achieved strong results across 3-fold cross-validation. The accuracies remained highly above 75%, reflecting good generalization and stable performance across folds. All Meta-models use a neural network with three hidden layers. Sensitivity and specificity were also well-balanced, indicating that the models maintained an appropriate trade-off between correctly identifying positive cases and limiting false alarms. Table 9 summarizes the 3-fold training performance metrics, and Table 10 summarizes the 3-fold test performance metrics; both tables align closely with the trends reported in the earlier training–testing evaluations.

**Figure 11:**
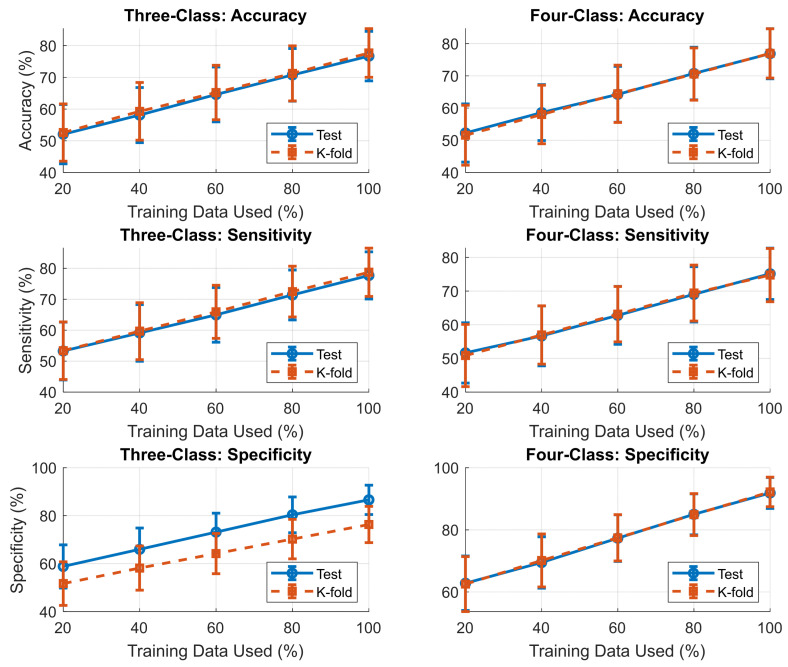
Learning curves for accuracy (**top**), sensitivity (**middle**), and specificity (**bottom**) for the three-class (**left**) and four-class (**right**) OSA severity models. Solid lines denote independent test performance and dashed lines denote k-fold cross-validation. All metrics improve monotonically with increasing training data and show no saturation, indicating a data-limited regime and consistent generalization across evaluation strategies.

To assess whether the proposed framework is limited by data availability, we conducted a learning-curve analysis by training the meta-model on progressively larger fractions of the training set (from 20% to 100%) while evaluating performance on a fixed held-out test set. As shown in Figure 11, test accuracy increased monotonically with training size for both the three-class and four-class configurations, without clear saturation. This trend suggests that current performance is primarily constrained by dataset size rather than model capacity, and that further gains are expected with larger, more diverse training cohorts.

Overall, the 3-fold cross-validation results confirm the reliability and robustness of the proposed approach across both the three-class and four-class classification frameworks. The OOB and test metrics demonstrate consistent performance, with strong sensitivity and specificity values that indicate reliable class discrimination and controlled false-positive rates. Table 11 provides a consolidated comparison of OOB, independent test, and k-fold cross-validation performance. Although OOB estimates are consistently optimistic, the close agreement between test and cross-validation results indicates stable generalization with minimal evaluation bias. Together with the conformal prediction coverage and set-size results, these findings demonstrate consistent performance across folds and robustness when transitioning from OOB estimates to independent testing, supporting the potential of the proposed framework for practical OSA severity assessment.

### 3.3. Statistical Significance Analysis

To evaluate whether the proposed meta-model significantly outperformed the six best base classifiers, we conducted paired statistical tests using the predictions and scores from the respective best-performing models. Rather than aggregating results across multiple trials, the tests were performed directly on the final model outputs obtained from the held-out test set. This approach allows for a direct comparison of model performance on the same set of samples. All statistical evaluations were based on the model predictions generated across the 3-fold cross-validation test sets, ensuring that the comparisons reflect consistent performance across multiple data splits rather than a single held-out partition.

Pairwise comparisons between the meta-model and each base classifier were conducted using both a parametric paired *t*-test and a non-parametric Wilcoxon signed-rank test applied to per-sample accuracy outcomes. These complementary tests assess the statistical significance of differences in continuous performance measures while accounting for possible violations of normality assumptions [26]. Additionally, McNemar’s test was employed to evaluate differences in binary classification decisions on a per-sample basis, providing insight into whether the models made significantly different classification errors [27]. These statistical comparisons were performed using the combined per-sample predictions obtained across all three folds of the cross-validation procedure.

To quantify the magnitude of observed differences, effect sizes were computed alongside *p*-values. Cohen’s d was calculated for the paired *t*-test results, and Cliff’s δ was derived from the Wilcoxon test outcomes. Furthermore, Bonferroni-adjusted *p*-values were used to control for the risk of false positives due to multiple comparisons across the six base classifiers, setting the adjusted significance threshold at α = 0.00833. The results demonstrate that, in all six comparisons, the meta-model significantly outperformed each base classifier, with *p*-values well below the adjusted threshold. Effect sizes were consistently large (Cohen’s d greater than 0.65 and Cliff’s δ at or above 0.36), indicating that the improvements are not only statistically significant but also practically meaningful. Table 12 shows the results of the statistical comparison.

## 4. Discussion

This study demonstrates the feasibility of multi-class OSA severity screening during wakefulness using tracheal breathing sounds and anthropometric features. The proposed meta-model achieved approximately 77% test accuracy across three- and four-class configurations, with high specificity and calibrated uncertainty estimates, supporting its potential role as a rapid screening and triage tool. The integration of meta-learning and conformal prediction contributes to the model’s robustness, interpretability, and potential for real-world clinical deployment. This discussion synthesizes the core findings, contextualizes them within physiological relevance, and evaluates implications for clinical applications.

### 4.1. Meta-Model Performance for Training-Testing

The meta-model was evaluated through a structured two-stage approach: out-of-bag (OOB) validation and blind testing on unseen data. In the three-class classification task, a moderate performance decline (from 91% to 76.7%) was observed during blind testing. This is commonly seen in ensemble learning due to overfitting or data variability [28,29]. However, the accuracy still remained within acceptable margin. Misclassifications primarily occurred between the Mild and Moderate classes, which share acoustic and physiological features, such as partial upper airway obstruction and variable inspiratory effort [15].

In the four-class classification, which includes the Severe category, the task became more challenging due to increased variability and finer-grained distinctions between classes. Misclassifications were most frequent between the Mild and Moderate groups, reflecting their overlapping acoustic and physiological characteristics, such as partial upper airway obstruction and variable inspiratory effort [28,29]. Despite the increased complexity, the models maintained meaningful discrimination across all four severity levels, highlighting their potential clinical utility. Comparatively, the three-class scenario, which combined Mild and Moderate cases into a single group, showed slightly higher overall accuracy, confirming that broader class definitions reduce ambiguity but may obscure clinically relevant distinctions.

Conformal prediction was incorporated to provide uncertainty quantification alongside severity stratification. The conformal models achieved the targeted statistical coverage level in both three-class and four-class configurations, with an average prediction set size of approximately two classes. In some instances, the resulting prediction sets contained multiple severity classes, indicating increased uncertainty in borderline samples rather than definitive clinical classification. This behavior is consistent with the theoretical properties of conformal prediction and supports transparent, uncertainty-aware screening. Rather than implying diagnostic certainty, these results demonstrate the feasibility of augmenting wakefulness-based OSA screening with calibrated confidence information to support cautious clinical triage. Visualizations of conformal prediction maps further illustrate class-dependent uncertainty patterns and regions of prediction ambiguity [30,31].

The use of stacked ensemble architecture allowed the model to integrate complementary strengths of diverse classifiers while reducing correlated error [28,32]. This architecture, combined with conformal prediction, enabled adaptive error correction and probabilistic confidence assignment, which are critical for medical decision support systems [33]. This work extends our previous binary-classification framework [18]. By integrating it into a unified, multi-class prediction model with conformal prediction to quantify model confidence, making it more suitable for clinical decision support. The stacking framework helped mitigate error propagation from base models, and the integration of conformal prediction further strengthened the model by providing uncertainty estimates for individual predictions, an essential attribute in clinical risk stratification. The stacking structure of the meta-model combining diverse, specialized base classifiers likely contributed to this robustness by reducing correlated errors and promoting generalization.

Detailed per-class analysis provides further insights into the model’s behavior. In the three-class task, the Non-OSA group achieved 81.8% sensitivity and 73.7% specificity, demonstrating strong identification of healthy individuals. The Mild-OSA group was detected with 80.0% sensitivity, 100% specificity, and the highest AUC of 0.93, indicating a conservative classification pattern that minimized false positives. This marks a significant improvement over earlier models, which frequently misclassified Mild cases [17,34]. The Moderate/Severe OSA group achieved 71.4% sensitivity and 87.5% specificity, reflecting residual difficulty in discriminating more severe cases due to overlapping acoustic characteristics and within-class variability [15,35].

In the four-class scenario, the Non-OSA and Mild OSA groups achieved sensitivities of 91% and 80%, with corresponding specificities of 84% and 96%, respectively. In contrast, sensitivity decreased for Moderate OSA (63%) and Severe OSA (68%), although both classes maintained high specificity values of 91% and 96%. These results indicate that the model consistently limits false-positive predictions across all severity levels, while reduced sensitivity in the Moderate and Severe classes likely reflects inter-class acoustic similarity and the impact of imbalanced data distribution [9,34,36].

Taken together, the three-class framework yielded more balanced sensitivity across groups, while the four-class division provided finer granularity but at the cost of reduced sensitivity for moderate and severe OSA. The preferred scheme may therefore depend on clinical priorities: if the goal is to minimize missed diagnoses, particularly in preoperative screening, the three-class grouping may be more practical. Conversely, when detailed stratification is required for treatment planning, the four-class scheme is more informative. It is also likely that with a larger and more balanced dataset, the performance gap between the two approaches would diminish, particularly for moderate and severe cases where class overlap and limited sample size currently constrain sensitivity.

While the proposed framework demonstrates high specificity and robust performance for wakefulness-based screening, reduced sensitivity for moderate and severe OSA indicates that it should not be used to exclude clinically significant disease. Nevertheless, the models sustained meaningful discrimination across all four severity levels, supporting their utility for rapid screening and triage rather than definitive diagnosis. Comparatively, the three-class configuration achieved slightly higher test accuracy, suggesting that broader class definitions reduce ambiguity, albeit at the expense of finer-grained clinical detail. Accordingly, positive or uncertain cases should be referred to for confirmatory overnight testing.

### 4.2. Meta-Model Performance for K-Fold

The meta-model was evaluated using a two-stage procedure consisting of out-of-bag (OOB) validation and blind testing on unseen data, supported by 3-fold cross-validation to ensure robust performance assessment. In the three-class classification task, the OOB accuracy remained high across folds, ranging from 82.2% to 87.9%, with balanced sensitivity and specificity levels (Table 9). During blind testing, accuracy decreased to values between 75.8% and 78.8% (Table 10), reflecting a moderate performance reduction (from 91% previously to 76.7%). This level of decline is commonly reported in ensemble and deep learning studies due to increased data variability and potential overfitting effects [20,21]. Despite this reduction, performance remained within acceptable ranges, and most misclassifications occurred between the Mild and Moderate groups—two categories known to share overlapping acoustic and physiological traits such as partial airway obstruction and variable inspiratory effort [9].

In the four-class configuration, which further separates Severe cases, the task understandably became more complex. OOB accuracies remained strong and consistent across folds (87.4–89.1%), again supported by well-balanced sensitivity and specificity values (Table 9). Blind testing results showed lower accuracies (75.6–77.4%), reflecting increased classification difficulty arising from finer granularity and wider intra-class variability (Table 10). Misclassifications were most frequent between the Mild and Moderate groups, reinforcing the physiological overlap between these categories [28,29].

To reinforce predictive reliability, conformal prediction was used to quantify per-sample uncertainty. Across all folds and in both class configurations, conformal prediction achieved full coverage (1.0) with an average prediction set size of 2, demonstrating the model’s high confidence and consistent uncertainty calibration. These outcomes were further supported by conformal prediction visualization maps, which helped identify class-dependent uncertainty structures and prediction boundaries [30,31].

The stacked ensemble architecture played a central role in achieving these results. By integrating the complementary strengths of diverse base classifiers and reducing correlated error, the ensemble improved both generalization and resilience across cross-validation folds [28,32]. The combined use of stacking and conformal prediction improved adaptive error correction and supported probabilistic confidence estimation—key requirements for clinical decision support [33]. Building on earlier binary classification work [18], this unified, multi-class framework offers enhanced explanatory value through uncertainty quantification and improved granularity in OSA severity assessment. The consistent 3-fold results shown in Table 9 and Table 10 underscore the robustness of the approach and support its potential for real-world clinical deployment.

The reported 95% confidence intervals allow a more conservative and transparent assessment of the proposed meta-model’s performance. Across both three-class and four-class classification tasks, OOB results exhibit consistently narrow confidence intervals, indicating stable ensemble behavior and reliable internal generalization. As expected, confidence intervals for the independent test results are wider due to the limited sample size; however, they remain well centered around the mean values and show no excessive dispersion, suggesting that the observed performance is not driven by isolated data splits. From a clinical perspective, the relatively tight confidence intervals for sensitivity are particularly important, as they indicate consistent identification of affected cases across repeated evaluations, reducing the risk of unstable diagnostic behavior. The overlap between OOB and test confidence intervals further supports the absence of substantial overfitting and demonstrates that the meta-model generalizes robustly to unseen data. Overall, the confidence interval analysis strengthens the reliability of the reported results by explicitly quantifying uncertainty while confirming the stability of the proposed approach.

### 4.3. Statistical Significance of Meta-Model Improvements

The statistical analyses conducted provide strong evidence that the meta-model offers a meaningful improvement over the individual base classifiers. By comparing predictions directly on the same test samples and using a combination of parametric, non-parametric, and categorical error tests [26,27,37]. We ensured a thorough and reliable evaluation of performance differences. All statistical comparisons were based on the combined predictions obtained across the 3-fold cross-validation test sets, ensuring that the results reflect consistent performance across multiple data splits rather than a single evaluation set. The consistent statistical significance across all tests, along with large effect sizes, indicates that the observed accuracy gains are not only unlikely to be due to chance but also practically important.

These findings highlight the benefit of the meta-model’s ensemble approach in effectively integrating diverse classifiers, mitigating individual model limitations, and improving overall predictive performance. The use of multiple complementary statistical evaluation methods further strengthens confidence in the robustness of the observed trends. Collectively, these results provide preliminary evidence supporting the meta-model’s potential value for OSA severity classification and suggest advantages over relying on any single base classifier within this proof-of-concept framework.

### 4.4. Comparison to Prior TBS Studies

A quantitative comparison between the proposed meta-model framework and representative prior studies on tracheal breathing sound–based OSA assessment is provided. To ensure a fair and informative comparison, we focus on key methodological and performance dimensions that are consistently reported across studies: dataset size, task formulation (binary vs. multi-class), evaluation performance (accuracy/AUC where available), recording condition (awake vs. sleep), and the presence of explicit uncertainty modeling. Only the top six prior works with clearly reported quantitative results and comparable awake TBS settings are highlighted. Table 13 summarizes a quantitative comparison between the proposed meta-model framework and representative prior tracheal breathing sound–based OSA studies. Unlike prior studies that predominantly address binary OSA screening without uncertainty quantification, the proposed method uniquely supports multi-class OSA severity stratification and integrates conformal prediction to provide statistically valid uncertainty estimates.

### 4.5. Clinical Utility and Limitations

The proposed OSA screening method offers several advantages over traditional overnight PSG testing. By utilizing short wakeful breathing recordings and easily obtainable anthropometric features, the model enables rapid screening, potentially under 10 min. This design is especially suitable for preoperative risk assessment, primary care triage, and telehealth settings where time and equipment are limited [4,7,39,40].

High specificity in detecting Non-OSA and Mild-OSA cases reduces unnecessary referrals for full PSG studies, thereby optimizing resource allocation. Meanwhile, sufficient sensitivity in identifying higher-risk individuals ensures critical cases are not overlooked. The model’s confidence-calibrated outputs via conformal prediction enhance its interpretability for clinicians and support risk-based decision-making [30,31,41,42].

Nonetheless, limitations remain. Misclassification was most prominent between the Mild and Moderate OSA classes, consistent with the results observed in the three-class scenario and highlighting the physiological and acoustic overlap between these groups. Extending the analysis to the four-class classification, which includes the Severe category, introduced additional complexity, with slightly lower accuracy due to finer-grained distinctions. Incorporating additional discriminative features such as those derived from deep learning models, breathing phase segmentation, or higher-resolution time–frequency representations could improve class separability [43,44,45,46,47]. Furthermore, as this study relied on a single dataset, external validation on independent cohorts is necessary to assess generalizability across clinical environments. To address this limitation, a new dataset is currently being collected under a separate acquisition protocol for future external validation. Until such validation is completed, the present findings should be interpreted as exploratory and proof-of-concept, rather than as evidence of clinical generalizability.

## 5. Conclusions

This study, for the first time, introduces a new, rapid, with acceptable accuracy meta-modeling framework for detecting and stratifying OSA severity into four clinically meaningful categories using TBS and anthropometric data recorded during wakefulness. The model achieved an average 76.7% test accuracy with high sensitivity and specificity across all severity levels. Leveraging stacked ensemble learning with robust base classifiers, the proposed approach offers a practical, PSG-free alternative for screening and early triage of OSA. A key contribution of this work is the development of a clinically motivated multi-class severity stratification framework tailored to wakefulness-based OSA assessment. The pairwise acoustic specialization strategy enables robust discrimination and aggregation across adjacent severity levels, effectively addressing the acoustic overlap inherent in progressive OSA severity. Furthermore, the integration of conformal prediction provides calibrated, sample-specific uncertainty estimates, directly addressing safety and reliability concerns associated with clinical deployment. Training on stratified anthropometric subgroups further improves interpretability, a crucial consideration for future regulatory approval. Its sub-10 min acquisition time, coupled with offline processing, makes it suitable for deployment in preoperative clinics, dental practices, and at-home settings. Compared to our previous binary-classification framework, this work expands the capabilities to a four-class system with reliability estimation, enabling finer severity stratification. Importantly, the proposed framework demonstrated consistent performance across 3-fold cross-validation, with OOB and blind test accuracies remaining stable in both the three-class and four-class configurations, supporting the model’s robustness and reducing the likelihood that the reported results are due to overfitting or dataset partitioning effects. Future work should focus on external validation using diverse populations and integrating additional physiological signals to improve generalizability and predictive performance further.

## Figures and Tables

**Table 1:** Severity groups and participants’ anthropometric information. AHI: apnea–hypopnea index (events/h), Sex (M/F): male/female, BMI: body mass index (kg/m^2^), MPS: Mallampati score (1/2/3/4), NC: neck circumference (cm).

Group	Num. of Subjects	AHI	Age	Sex	BMI	MPS	NC
Non-OSA	74	1.18 ± 1.26	46.84 ± 12.89	29 M, 45 F	30.61 ± 6.29	41(1), 19(2), 6(3), 8(4)	38.78 ± 4.04
Mild-OSA	35	8.69 ± 2.58	52.31 ± 11.57	21 M, 14 F	34.29 ± 8.41	18(1), 6(2), 9(3), 1(4)	42.09 ± 6.49
Moderate-OSA	50	21.51 ± 4.23	54.74 ± 11.33	36 M, 14 F	33.78 ± 6.39	17(1), 17(2), 8(3), 8(4)	43.10 ± 3.43
Severe-OSA	40	69.54 ± 33.26	48.98 ± 11.15	30 M,10 F	39.75 ± 8.65	5(1), 13(2), 14(3), 8(4)	45.28 ± 3.64

**Table 2:** Severity groups and Participants’ anthropometric information of the training set. AHI: apnea–hypopnea index (events/h), Sex (M/F): male/female, BMI: body mass index (kg/m^2^), MPS: Mallampati score (1/2/3/4), NC: neck circumference (cm).

Group	Num. of Subjects	AHI	Age	Sex	BMI	MPS	NC
Non-OSA	63	1.2 ± 1.2	46.8 ± 13.5	24 M, 39 F	30.6 ± 6.6	36 (1), 17 (2), 5 (3), 5 (4)	39.0 ± 3.5
Mild-OSA	30	8.8 ± 2.6	50.9 ± 10.4	17 M, 13 F	34.1 ± 8.2	14 (1), 7 (2), 8 (3), 0 (4)	41.5 ± 7.0
Moderate-OSA	42	22.0 ± 4.3	53.7 ± 10.3	31 M, 11 F	33.7 ± 6.8	11 (1), 15 (2), 8 (3), 8 (4)	43.3 ± 3.3
Severe-OSA	34	68.5 ± 32.5	50.9 ± 11.1	24 M, 10 F	39.9 ± 9.3	4 (1), 11 (2), 13 (3), 6 (4)	45.0 ± 3.4

**Table 3:** Severity groups and Participants’ anthropometric information of the test set. AHI: apnea–hypopnea index (events/h), Sex (M/F): male/female, BMI: body mass index (kg/m^2^), MPS: Mallampati score (1/2/3/4), NC: neck circumference (cm).

Group	Num. of Subjects	AHI	Age	Sex	BMI	MPS	NC
Non-OSA	11	0.9 ± 1.0	45.4 ± 10.7	6 M, 5 F	30.6 ± 3.8	6 (1), 3 (2), 1 (3), 1 (4)	40.1 ± 4.5
Mild-OSA	5	6.7 ± 1.7	52.4 ± 12.6	3 M, 2 F	30.3 ± 10.2	3 (1),1 (2), 1 (3), 0 (4)	41.9 ± 1.5
Moderate-OSA	8	20.7 ± 4.5	58.5 ± 7.3	4 M, 4 F	33.7 ± 6.2	4 (1), 3 (2), 1 (3), 0 (4)	41.5 ± 4.0
Severe-OSA	6	80.0 ± 35.1	46.4 ± 11.3	4 M, 2 F	44.4 ± 6.2	1 (1), 2 (2), 1 (3), 2 (4)	44.0 ± 3.7

**Table 4:** Demographic, anthropometric, and clinical characteristics of participants across OSA severity groups and cross-validation folds. Values are reported as mean ± standard deviation. Each row corresponds to one fold within a given severity group, illustrating the balance of subject distribution and key confounding variables across folds. AHI: apnea–hypopnea index (events/h), Sex (M/F): male/female, BMI: body mass index (kg/m^2^), MPS: Mallampati score (1/2/3/4), NC: neck circumference (cm).

Group (Fold)	Num. of Subjects	AHI	Age	Sex (M/F)	BMI	MPS	NC
Non-OSA (F1)	23	0.6 ± 0.8	44.9 ± 12.1	10/13	29.2 ± 4.7	12, 7, 1, 3	38.0 ± 4.7
Non-OSA (F2)	27	1.1 ± 1.3	45.7 ± 12.1	10/17	32.3 ± 7.6	12, 9, 4, 2	39.2 ± 4.3
Non-OSA (F3)	24	1.8 ± 1.3	50.0 ± 14.3	9/15	30.0 ± 5.8	17, 3, 1, 3	39.0 ± 2.9
Mild-OSA (F1)	16	8.7 ± 2.4	50.9 ± 12.5	10/6	36.6 ± 9.9	7, 1, 7, 1	43.5 ± 5.3
Mild-OSA (F2)	10	8.6 ± 2.2	51.3 ± 11.6	5/5	31.8 ± 8.4	6, 1, 2, 1	38.5 ± 8.8
Mild-OSA (F3)	9	8.8 ± 3.5	56.0 ± 10.3	6/3	33.0 ± 4.2	5, 4, 0, 0	43.4 ± 2.7
Moderate-OSA (F1)	16	19.9 ± 2.9	56.3 ± 10.8	10/6	34.6 ± 7.8	4, 6, 2, 4	42.3 ± 4.0
Moderate-OSA (F2)	18	22.8 ± 4.5	53.6 ± 9.7	13/5	31.8 ± 5.7	8, 5, 3, 2	42.7 ± 3.8
Moderate-OSA (F3)	16	21.6 ± 4.7	54.5 ± 13.9	13/3	35.2 ± 5.2	5, 6, 3, 2	43.6 ± 2.8
Severe-OSA (F1)	14	72.9 ± 35.0	45.5 ± 10.5	11/3	39.1 ± 10.1	2, 3, 5, 4	44.3 ± 4.2
Severe-OSA (F2)	16	66.6 ± 29.6	50.2 ± 11.0	13/3	40.1 ± 8.5	1, 6, 5, 4	46.6 ± 3.2
Severe-OSA (F3)	10	69.6 ± 39.1	51.9 ± 12.2	6/4	40.2 ± 7.5	2, 4, 4, 0	43.8 ± 3.5

**Table 5:** Results for the Meta-Model for three classes (mean ± 95% CI).

Metric	Accuracy		Sensitivity	Specificity
Meta-Model Model (Neural Net)	OOB Results			
	91.1 ± 1.8		91.6 ± 1.7	95.3 ± 1.5
	Test Results			
	76.7 ± 2.6		77.7 ± 2.5	87.1 ± 2.3
Conformal Results	Coverage	1	Avg Set Size	2

**Table 6:** Results for the Meta-Model for four classes (mean ± 95% CI).

Metric	Accuracy		Sensitivity	Specificity
Meta-Model Model (Neural Net)	OOB Results			
	88.2 ± 2.0		91.6 ± 1.9	88.2 ± 2.0
	Test Results			
	76.7 ± 2.7		75 ± 2.6	92 ± 2.1
Conformal Results	Coverage	1	Avg Set Size	2

**Table 7:** Results for the Meta-Model for three classes.

Metric	Accuracy	Sensitivity	Specificity
Non-OSA	76.7	81.8	73.7
Mild-OSA		80.0	100
Moderate/Severe-OSA		71.4	87.5

**Table 8:** Results for the Meta-Model for four classes.

Metric	Accuracy	Sensitivity	Specificity
Non-OSA	76.7	91	84
Mild-OSA		80	96
Moderate-OSA		63	91
Severe-OSA		68	96

**Table 9:** Train 3-fold cross-validation results of experimental models.

Experiment	Fold	Accuracy	Sensitivity	Specificity
Three Classes	1	82.2	83.1	80.7
	2	87.9	87.8	88
	3	86.0	84.3	86.3
Mean ± Standard Deviation		85.4 ± 2.9	85.1 ± 2.4	85 ± 3.8
Conformal Results	Coverage	1	Avg Set Size	2
Four Classes	1	87.4	90.8	87.9
	2	89.1	92.3	88.4
	3	88.2	91.7	88.3
Mean ± Standard Deviation		88.2 ± 0.9	91.6 ± 0.8	88.2 ± 0.3
Conformal Results	Coverage	1	Avg Set Size	2

**Table 10:** Test 3-fold cross-validation results of experimental models.

Experiment	Fold	Accuracy	Sensitivity	Specificity
Three Classes	1	78.7	80.4	76.9
	2	78.8	79.2	78.2
	3	75.8	76.2	74.1
Mean ± Standard Deviation		77.8 ± 1.7	78.6 ± 2.2	76.4 ± 2.1
Conformal Results	Coverage	1	Avg Set Size	2
Four Classes	1	77.1	75.8	91.0
	2	75.6	74.4	93.2
	3	77.4	74.8	91.9
Mean ± Standard Deviation		76.7 ± 1.0	75 ± 0.7	92 ± 1.1
Conformal Results	Coverage	1	Avg Set Size	2

**Table 11:** Summary comparison of OOB, independent test, and 3-fold cross-validation mean performance for the proposed meta-model.

Classes	Evaluation	Accuracy (%)	Sensitivity (%)	Specificity (%)
Three	OOB	91.1 ± 1.8	91.6 ± 1.7	95.3 ± 1.5
Three	Test	76.7 ± 2.6	77.7 ± 2.5	87.1 ± 2.3
Three	3-fold Train CV (Mean ± SD)	85.4 ± 2.9	85.1 ± 2.4	85.0 ± 3.8
Three	3-fold Test CV (Mean ± SD)	77.8 ± 1.7	78.6 ± 2.2	76.4 ± 2.1
Four	OOB	88.2 ± 2.0	91.6 ± 1.9	88.2 ± 2.0
Four	Test	76.7 ± 2.7	75.0 ± 2.6	92.0 ± 2.1
Four	3-fold Train CV (Mean ± SD)	88.2 ± 0.9	91.6 ± 0.8	88.2 ± 0.3
Four	3-fold Test CV (Mean ± SD)	76.7 ± 1.0	75.0 ± 0.7	92.0 ± 1.1

**Table 12:** Statistical significance analysis comparing the proposed meta-model to the base classifier.

Comparison	Paired *t*-Test (ρ)	Wilcoxon (ρ)	McNemar (ρ)	Mean Δ Accuracy	Cohens’ (d)	Cliffs (δ)	Bonferroni (ρ)
Meta-model vs. Base-model 1	1.10 × 10^−3^	3.40 × 10^−3^	3.40 × 10^−3^	0.3667	0.6594	0.3667	1.10 × 10^−3^
Meta-model vs. Base-model 2	3.07 × 10^−4^	9.77 × 10^−4^	9.77 × 10^−4^	0.3667	0.7481	0.3667	3.07 × 10^−4^
Meta-model vs. Base-model 3	1.09 × 10^−4^	4.65 × 10^−4^	5.19 × 10^−4^	0.4667	0.8168	0.4667	1.09 × 10^−4^
Meta-model vs. Base-model 4	8.70 × 10^−6^	6.10 × 10^−5^	6.10 × 10^−5^	0.5	0.9832	0.5	8.70 × 10^−6^
Meta-model vs. Base-model 5	1.85 × 10^−5^	1.62 × 10^−4^	1.45 × 10^−4^	0.5333	0.9335	0.5333	1.85 × 10^−5^
Meta-model vs. Base-model 6	4.61 × 10^−5^	2.75 × 10^−4^	2.75 × 10^−4^	0.5	0.8736	0.5	4.61 × 10^−5^
Bonferroni-corrected alpha	0.00833						

A ρ-value < 0.05 indicates a statistically significant difference.

**Table 13:** Quantitative comparison of the proposed meta-model framework with representative prior TBS–based OSA studies in terms of dataset size, task formulation, performance, recording condition, and uncertainty modeling.

Study	Dataset Size	Task	Performance Metrics	Uncertainty Modeling
[8]	109 (AHI < 15) 90 (AHI > 15)	Binary	Test: 81.4% accuracy, 80.9% sensitivity, 82.1% specificity:	No
[15]	61 (AHI < 5) 69 (AHI > 10)	Binary	Validation: 84.5% accuracy 88.2% sensitivity, 80.9%specificity	No
[17]	109 (AHI < 15) 90 (AHI > 15)	Binary	79.3% accuracy, 82.2% sensitivity, 75.8% specificity	No
[34]	109 (AHI < 15) 90 (AHI > 15)	Binary	RF outperformed LR by +3.5% accuracy (~82–85% range)	No
[38]	43 OSA 47 controls	Binary	76.5% accuracy, 55% sensitivity, 100% specificity	No
This Work	(74 non-OSA, 35 Mild-OSA, 90 Moderate/Severe-OSA)	Multi-class (3 classes)	Test: 76.7% accuracy, 77.7% sensitivity, 87.1% specificity 3-fold OOB: 82.2–87.9% accuracy, 83.1–87.8% sensitivity, 80.7–88.0% specificity 3-fold Test: 75.8–78.8% accuracy, 76.2–79.2% sensitivity, 74.1–78.2% specificity	Yes (Conformal Prediction)
This Work	(74 non-OSA, 35 Mild-OSA, 50 Moderate-OSA, and 40 Severe-OSA)	Multi-class (4 classes)	Test: 76.7% accuracy, 75% sensitivity, 92% specificity 3-fold OOB: 87.4–89.1% accuracy, 90.8–92.3% sensitivity, 87.9–88.4% specificity 3-fold Test: 75.6–77.4% accuracy, 74.4–75.8% sensitivity, 91.0–93.2% specificity	Yes (Conformal Prediction)

## Data Availability

The data presented in this study are available on request from the PI of the study (last author) due to ethical and consent-related restrictions, as the datasets contain human audio recordings and can only be shared under controlled access following approval of a signed data-use consent agreement.

## Abbreviations

The following abbreviations are used in this manuscript:

AHI	Apnea–Hypopnea Index
BMI	Body Mass Index
CQT	Constant-Q Transform
CP	Conformal Prediction
DFA	Detrended Fluctuation Analysis
ECOC	Error-Correcting Output Codes
GBM	Gradient Boosting Machine
kNN	k-Nearest Neighbors
LASSO	Least Absolute Shrinkage and Selection Operator
LDA	Linear Discriminant Analysis
LR	Logistic Regression
MFCC	Mel-Frequency Cepstral Coefficients
MPS	Mallampati Score
NC	Neck Circumference
OOB	Out-of-Bag
OSA	Obstructive Sleep Apnea
PSG	Polysomnography
RF	Random Forest
RFE	Recursive Feature Elimination
RQA	Recurrence Quantification Analysis
SHAP	SHapley Additive exPlanations
SVM	Support Vector Machine
TBS	Tracheal Breathing Sounds
TQWT	Tunable Q-Factor Wavelet Transform
VMD	Variational Mode Decomposition

## References

[1] Rizzo D., Baltzan M., Sirpal S., Dosman J., Kaminska M., Chung F. (2024). Prevalence and Regional Distribution of Obstructive Sleep Apnea in Canada: Analysis from the Canadian Longitudinal Study on Aging. Can. J. Public Health.

[2] Faria A., Allen A.H., Fox N., Ayas N., Laher I. (2021). The Public Health Burden of Obstructive Sleep Apnea. Sleep. Sci..

[3] American Academy of Sleep Medicine (2016). Hidden Health Crisis Costing America Billions: Underdiagnosing and Undertreating Obstructive Sleep Apnea Draining Healthcare System.

[4] Kushida C.A., Littner M.R., Morgenthaler T., Alessi C.A., Bailey D., Coleman J., Friedman L., Hirshkowitz M., Kapen S., Kramer M. (2005). Practice Parameters for the Indications for Polysomnography and Related Procedures: An Update for 2005. Sleep.

[5] Chung F., Liao P., Elsaid H., Islam S., Shapiro C.M., Sun Y. (2012). Oxygen Desaturation Index from Nocturnal Oximetry. Anesth. Analg..

[6] Hoang N.H., Liang Z. (2025). Detection and Severity Classification of Sleep Apnea Using Continuous Wearable SpO_2_ Signals: A Multi-Scale Feature Approach. Sensors.

[7] Corral-Peñafiel J., Pepin J.-L., Barbe F. (2013). Ambulatory Monitoring in the Diagnosis and Management of Obstructive Sleep Apnoea Syndrome. Eur. Respir. Rev..

[8] Elwali A., Moussavi Z. (2019). A Novel Decision Making Procedure during Wakefulness for Screening Obstructive Sleep Apnea Using Anthropometric Information and Tracheal Breathing Sounds. Sci. Rep..

[9] Montazeri A., Giannouli E., Moussavi Z. (2012). Assessment of Obstructive Sleep Apnea and Its Severity during Wakefulness. Ann. Biomed. Eng..

[10] Singh M., Liao P., Kobah S., Wijeysundera D.N., Shapiro C., Chung F. (2013). Proportion of Surgical Patients with Undiagnosed Obstructive Sleep Apnoea. Br. J. Anaesth..

[11] Chen L., Pivetta B., Nagappa M., Saripella A., Islam S., Englesakis M., Chung F. (2021). Validation of the STOP-Bang Questionnaire for Screening of Obstructive Sleep Apnea in the General Population and Commercial Drivers: A Systematic Review and Meta-Analysis. Sleep Breath..

[12] Behar J.A., Palmius N., Daly J., Li Q., Rizzatti F.G., Bittencourt L., Clifford G.D. Sleep Questionnaires in Screening for Obstructive Sleep Apnoea. Proceedings of the 2017 Computing in Cardiology.

[13] El-Sayed I.H. (2012). Comparison of Four Sleep Questionnaires for Screening Obstructive Sleep Apnea. Egypt. J. Chest Dis. Tuberc..

[14] Pataka A., Daskalopoulou E., Kalamaras G., Fekete Passa K., Argyropoulou P. (2014). Evaluation of Five Different Questionnaires for Assessing Sleep Apnea Syndrome in a Sleep Clinic. Sleep. Med..

[15] Elwali A., Moussavi Z. (2016). Obstructive Sleep Apnea Screening and Airway Structure Characterization During Wakefulness Using Tracheal Breathing Sounds. Ann. Biomed. Eng..

[16] Simply R.M., Dafna E., Zigel Y. (2020). Diagnosis of Obstructive Sleep Apnea Using Speech Signals From Awake Subjects. IEEE J. Sel. Top. Signal Process.

[17] Hajipour F., Jozani M.J., Elwali A., Moussavi Z. (2019). Regularized Logistic Regression for Obstructive Sleep Apnea Screening during Wakefulness Using Daytime Tracheal Breathing Sounds and Anthropometric Information. Med. Biol. Eng. Comput..

[18] Alqudah A.M., Moussavi Z. (2025). Assessing Obstructive Sleep Apnea Severity During Wakefulness via Tracheal Breathing Sound Analysis. Sensors.

[19] Brusa E., Cibrario L., Delprete C., Di Maggio L.G. (2023). Explainable AI for Machine Fault Diagnosis: Understanding Features’ Contribution in Machine Learning Models for Industrial Condition Monitoring. Appl. Sci..

[20] Wolpert D.H. (1992). Stacked Generalization. Neural Netw..

[21] Leblanc M., Tibshirani R. (1996). Combining Estimates in Regression and Classification. J. Am. Stat. Assoc..

[22] Shafer G., Vovk V. (2008). A Tutorial on Conformal Prediction. J. Mach. Learn. Res..

[23] Romano Y., Sesia M., Candès E.J. Classification with Valid and Adaptive Coverage. Proceedings of the 34th Conference on Neural Information Processing Systems (NeurIPS 2020).

[24] Gammerman A., Shafer G., Vovk V. (2005). Algorithmic Learning in a Random World.

[25] Rainio O., Teuho J., Klén R. (2024). Evaluation Metrics and Statistical Tests for Machine Learning. Sci. Rep..

[26] Demšar J. (2006). Statistical Comparisons of Classifiers over Multiple Data Sets. J. Mach. Learn. Res..

[27] Dietterich T.G. (1998). Approximate Statistical Tests for Comparing Supervised Classification Learning Algorithms. Neural Comput..

[28] Smyth P., Wolpert D. Stacked density estimation. Proceedings of the Advances in Neural Information Processing Systems.

[29] Japkowicz N., Stephen S. (2002). The Class Imbalance Problem: A Systematic Study. Intell. Data Anal..

[30] Berry R.B., Brooks R., Gamaldo C.E., Harding S.M., Lloyd R.M., Marcus C.L., Vaughn B.V. (2012). The AASM Manual for the Scoring of Sleep and Associated Events: Rules, Terminology and Technical Specifications.

[31] Espiritu J.R.D. (2021). Health Consequences of Obstructive Sleep Apnea. Management of Obstructive Sleep Apnea.

[32] Wang M., Qian Y., Yang Y., Chen H., Rao W.-F. (2024). Improved Stacking Ensemble Learning Based on Feature Selection to Accurately Predict Warfarin Dose. Front. Cardiovasc. Med..

[33] Sarker I.H. (2021). Machine Learning: Algorithms, Real-World Applications and Research Directions. SN Comput. Sci..

[34] Hajipour F., Jozani M.J., Moussavi Z. (2020). A Comparison of Regularized Logistic Regression and Random Forest Machine Learning Models for Daytime Diagnosis of Obstructive Sleep Apnea. Med. Biol. Eng. Comput..

[35] Alqudah A.M., Elwali A., Kupiak B., Hajipour F., Jacobson N., Moussavi Z. (2024). Obstructive Sleep Apnea Detection during Wakefulness: A Comprehensive Methodological Review. Med. Biol. Eng. Comput..

[36] He H., Garcia E.A. (2009). Learning from Imbalanced Data. IEEE Trans. Knowl. Data Eng..

[37] Gonçalves M.C., Silva R., Naldi M.C., Bianchi R.A.C. (2023). The Effect of Statistical Hypothesis Testing on Machine Learning Model Selection. Intelligent Systems (BRACIS 2023), Proceedings of the 12th Brazilian Conference, Belo Horizonte, Brazil, 25–29 September 2023.

[38] Simply R.M., Dafna E., Zigel Y. (2018). Obstructive Sleep Apnea (OSA) Classification Using Analysis of Breathing Sounds During Speech. Proceedings of the 2018 26th European Signal Processing Conference (EUSIPCO), Rome, Italy, 3–7 September 2018.

[39] Walia R., Achilefu A., Crawford S., Jain V., Wigley S.D., McCarthy L.H. (2014). Are At-Home Sleep Studies Performed Using Portable Monitors (PMs) as Effective at Diagnosing Obstructive Sleep Apnea (OSA) in Adults as Sleep Laboratory-Based Polysomnography (PSG)?. J. Okla. State Med. Assoc..

[40] Gali B., Whalen F.X., Gay P.C., Olson E.J., Schroeder D.R., Plevak D.J., Morgenthaler T.I. (2007). Management Plan to Reduce Risks in Perioperative Care of Patients with Presumed Obstructive Sleep Apnea Syndrome. J. Clin. Sleep Med..

[41] Van den Eynde J., Lachmann M., Laugwitz K.-L., Manlhiot C., Kutty S. (2023). Successfully Implemented Artificial Intelligence and Machine Learning Applications in Cardiology: State-of-the-Art Review. Trends Cardiovasc. Med..

[42] Begley T., Schwedes T., Frye C., Feige I. (2020). Explainability for Fair Machine Learning. arXiv.

[43] DeYoung P.N., Bakker J.P., Sands S.A., Batool-Anwar S., Connolly J.G., Butler J.P., Malhotra A. (2013). Acoustic Pharyngometry Measurement of Minimal Cross-Sectional Airway Area Is a Significant Independent Predictor of Moderate-to-Severe Obstructive Sleep Apnea. J. Clin. Sleep Med..

[44] Dae G.J., Hae Y.C., Grunstein R.R., Yee B. (2004). Predictive Value of Kushida Index and Acoustic Pharyngometry for the Evaluation of Upper Airway in Subjects with or without Obstructive Sleep Apnea. J. Korean Med. Sci..

[45] Vashishth T.K., Sharma V., Neha, Ahamad S. (2025). Exploring Machine Learning Models for Biomedical Signal Processing: A Comprehensive Review. Machine Learning Models and Architectures for Biomedical Signal Processing.

[46] Miotto R., Wang F., Wang S., Jiang X., Dudley J.T. (2018). Deep Learning for Healthcare: Review, Opportunities and Challenges. Brief. Bioinform..

[47] Ben Or D., Dafna E., Tarasiuk A., Zigel Y. (2016). Obstructive Sleep Apnea Severity Estimation: Fusion of Speech-Based Systems. EMBS 2016, Proceedings of the Annual International Conference of the IEEE Engineering in Medicine and Biology Society, Orlando, FL, USA, 16–20 August 2016.

